# Appetite measures during ketamine administration in treatment-resistant mood disorders: a naturalistic study

**DOI:** 10.3389/fnut.2026.1858899

**Published:** 2026-08-21

**Authors:** Agnieszka Mechlińska, Mariusz Wiglusz, Jakub Słupski, Krzysztof Pastuszak, Wiesław Jerzy Cubała

**Affiliations:** 1 Department of Psychiatry, Faculty of Medicine, Medical University of Gdańsk, Gdańsk, Poland; 2 Department of Algorithms and Systems Modelling, Gdańsk University of Technology, Gdańsk, Poland; 3 Laboratory of Translational Oncology, Intercollegiate Faculty of Biotechnology of the University of Gdańsk and the Medical University of Gdańsk, Gdańsk, Poland; 4 Center of Biostatistics and Bioinformatics, Medical University of Gdańsk, Gdańsk, Poland

**Keywords:** appetite, bipolar disorder, depression, ketamine, treatment-resistant mood disorders

## Abstract

This study evaluated the acute and short-term effects of repeated ketamine treatment on appetite in adult inpatients with treatment-resistant mood disorders, including major depressive disorder and bipolar disorder. Appetite, hunger, and desire to eat were assessed using the Visual Analog Scales (VAS) and the Simplified Nutritional Appetite Questionnaire (SNAQ) at baseline and at multiple time points up to 7 days following ketamine administration. No significant changes were observed over the course of treatment, and results were consistent across diagnostic groups and body mass index (BMI) categories. No increased risk of appetite-related undernutrition or clinically relevant weight loss was identified. These findings do not suggest a substantial short-term impact of ketamine on appetite regulation or body weight and are consistent with a favorable metabolic safety profile, supporting its clinical use in patients with treatment-resistant depression (TRD) and treatment-resistant bipolar disorder (TRBD).

## 1 Introduction

Treatment-resistant mood disorders affect approximately 100 million people worldwide and constitute a major clinical and socioeconomic burden, associated with severe functional impairment, disability, and increased suicide risk. Management remains challenging due to marked clinical heterogeneity and reliance on limited pharmacological options tailored to illness subtype (1–3).

A growing body of evidence indicates that obesity frequently co-occurs with depressive disorders and is associated with approximately 30%–40% higher risk of depression, likely reflecting shared neurobiological, hormonal, immune, and metabolic disturbances (4). This association is particularly salient in TRD and TRBD, which are often accompanied not only by obesity itself but also by related cardiometabolic conditions, including cardiovascular disease, metabolic syndrome, MASLD, and type 2 diabetes, thereby amplifying overall disease burden (1, 5). Population studies indicate that adverse metabolic and inflammatory profiles are associated with increased vulnerability to mood and stress-related disorders as well as greater cognitive impairment, underscoring the role of metabolic–inflammatory pathways in neuropsychiatric and neurocognitive dysfunction (6, 7).

Disturbances in appetite and eating behavior represent core features of mood disorders and contribute to a bidirectional relationship between depression and obesity, driven by shared dysregulation of rewards pathways, metabolic signaling, and chronic inflammation (8, 9). In addition, psychopharmacological treatment may further influence appetite and body weight through central and peripheral mechanisms affecting satiety, rewards processing, and metabolism (10, 11). Given the complex interplay between metabolic dysfunction and treatment resistance, novel interventions require careful evaluation of their metabolic effects. Appetite regulation depends on the interaction between central neurotransmitter systems and peripheral metabolic signals, including leptin, a hormone produced primarily by adipose tissue that plays a key role in satiety signaling and energy balance. Altered leptin levels and leptin resistance have been linked to obesity and atypical depressive symptoms, particularly increased appetite and weight gain (12). Experimental and clinical evidence further suggests that leptin signaling may influence antidepressant response, supporting a growing interest in metabolic pathways as potential contributors to mood disorders and treatment outcomes.

The increasing prevalence of treatment-resistant depression has highlighted the urgent need for novel therapeutic strategies, prompting increasing interest in ketamine as a rapid-acting antidepressant option. In contrast to conventional antidepressants, ketamine may improve depressive symptoms within hours of administration and has been linked to rapid reductions in suicidal ideation (13). Its antidepressant effects are thought to involve glutamatergic neurotransmission and neuroplasticity-related processes, including NMDA receptor blockade, enhanced AMPA signaling, activation of the mTOR pathway, and increased BDNF-TrkB activity (14). Ketamine may also affect inflammatory and metabolic pathways implicated in mood disorders (15). In contrast to its consistent impact on mood, its effects on appetite remain unclear and appear to vary across individuals. Current studies yield no convergent conclusions, reporting increases, decreases, or no measurable changes in appetite, highlighting the need for further investigation (16).

The aim of this study was to evaluate the acute and short-term effects of repeated ketamine treatment on appetite measures in patients with treatment-resistant mood disorders, with particular attention to changes in hunger, desire to eat, and overall appetite regulation during the course of therapy. In addition, we examined whether the response to ketamine differed by diagnostic category, mood improvement and BMI.

## 2 Materials and methods

### 2.1 Participants

The study was conducted at the Department of Psychiatry of the Medical University of Gdańsk (Poland) and included adult inpatients referred for short-term ketamine treatment. Participants were aged ≥ 18 years and met DSM-5 criteria for a treatment-resistant mood disorders, defined as an inadequate response to at least two appropriately dosed and adequately timed pharmacological treatments, including MDD and BD. All participants provided written informed consent prior to enrolment. Exclusion criteria included active malignancy, pregnancy, inability to cooperate, unstable medical condition, or refusal to undergo study procedures. Each participant completed a study protocol lasting approximately 5–6 weeks. The treatment phase lasted 4 weeks and involved twice-weekly ketamine administration. Body weight and height were collected to calculate BMI, classified according to World Health Organization (WHO) criteria. The study was registered on ClinicalTrials.gov (NCT04226963), approved by the Independent Bioethics Committee for Scientific Research at the Medical University of Gdańsk (NKBBN/172–674/2019) and conducted in accordance with the Declaration of Helsinki.

### 2.2 Appetite measures

**FIGURE 1:**
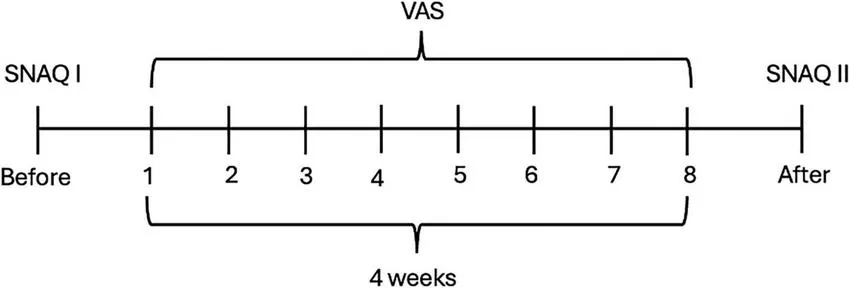
Study assessment protocol. Simplified Nutritional Appetite Questionnaire (SNAQ) was administered at baseline (SNAQ I) and after completion of the treatment protocol (SNAQ II). Visual Analog Scales (VAS) assessing appetite-related measures were collected repeatedly across eight ketamine administrations conducted over a 4-week period.

**FIGURE 2:**
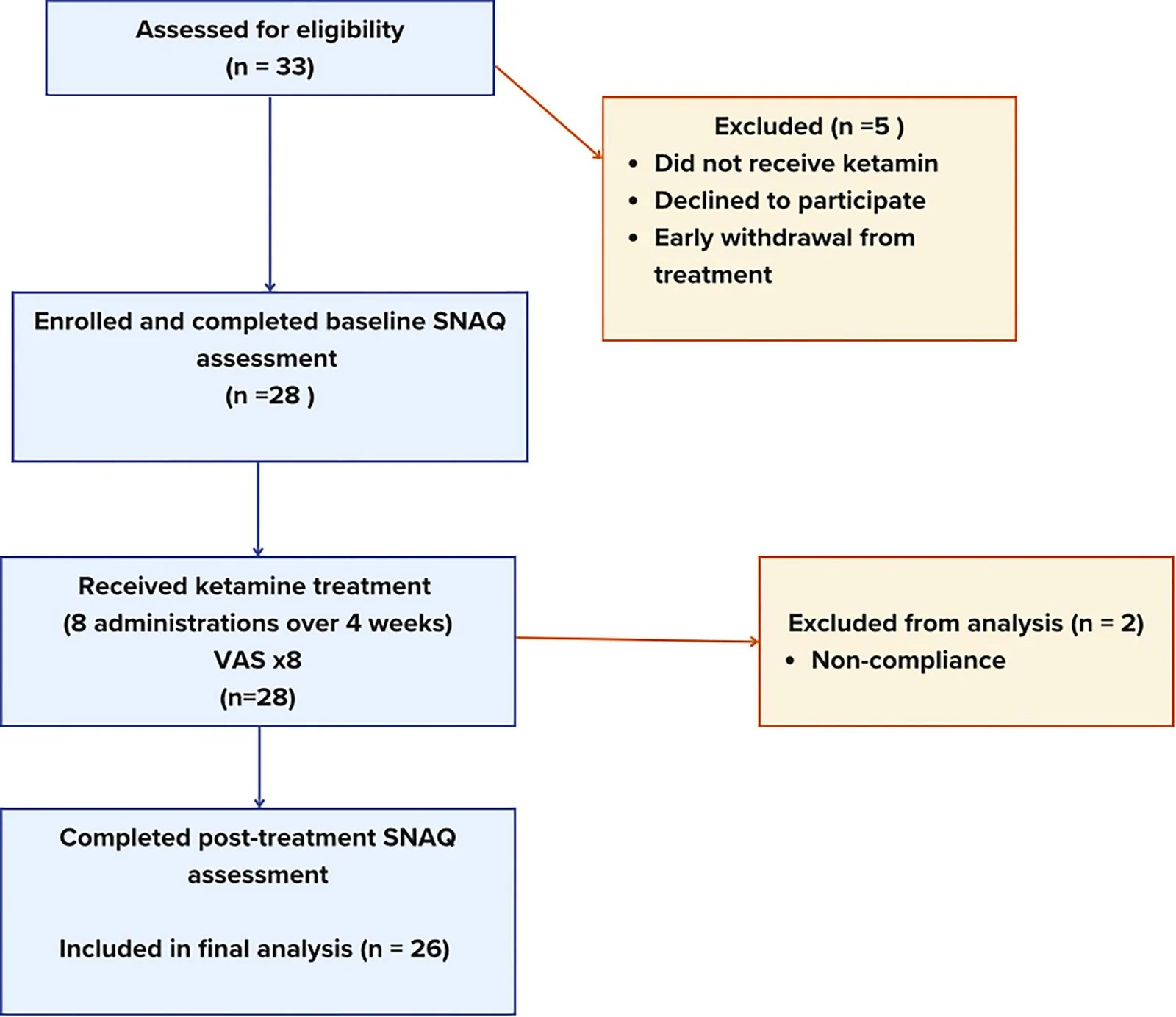
Participant flow diagram.

Appetite was assessed using two validated instruments: the Visual Analog Scales (VAS) and the Simplified Nutritional Appetite Questionnaire (SNAQ) (17, 18). The SNAQ evaluates appetite-related domains, including overall appetite, early satiety, perceived taste of food, and usual meal size, with total scores ranging from 0 to 20; a score of ≤14 indicates a clinically relevant risk of ≥5% body weight loss within 6 months. The VAS comprised three items assessing subjective appetite, hunger, and desire to eat. Responses were recorded on 10-cm horizontal lines with anchors at 0 (not at all) and 10 (extremely strong) for each dimension at predefined time points: prior to ketamine administration, and at 4, 8, 12, 24, and 48 h, as well as 7 days post-dose. This assessment schedule was applied consistently across all ketamine sessions. The overall measurement structure is illustrated in Figure 1. Figure 2 presents the participant recruitment process and study flow.

### 2.3 Patients’ diet

During hospitalization, patients received standard hospital meals and were not placed on a calorie-restricted diet. Several dietary options were available, including standard, easily digestible, vegetarian, and diabetic diets. Patients were allowed to choose and modify their dietary preferences throughout treatment according to their individual needs. All diets consisted of three daily meals (breakfast, lunch, and dinner). Patients also had access to snacks between meals without restrictions. No specific dietary intervention was implemented during the study to avoid influencing appetite-related outcomes.

### 2.4 Statistical analysis

Statistical analyses were performed using R version 4.3.2, with a significance threshold of *p* < 0.05. Categorical variables were compared using Fisher’s exact test, with the Freeman-Halton extension applied for variables with more than two levels. Numerical variables were assessed for normality using the Shapiro-Wilk test; normally distributed data were analyzed with paired *t*-tests (Welch’s correction applied if variances were unequal), while non-normal data were evaluated using paired Wilcoxon signed-rank tests. Given the exploratory nature of the study, no false discovery rate (FDR) correction was applied. Results are reported as mean (standard deviation) for normally distributed data or median (interquartile range) for non-normal data.

Additional exploratory analyses compared appetite-related changes between ketamine responders and non-responders. Clinical response was defined as a ≥ 50% reduction in MADRS total score from baseline to the last valid post-baseline assessment. Analyses were performed using complete cases for each outcome, without imputation. Change scores were calculated as post-treatment minus baseline values for SNAQ and as mean post-dose minus mean pre-dose ratings for VAS measures. Between-group differences were assessed using Welch’s *t*-tests, with Cohen’s d reported as the effect size. Associations between MADRS percentage improvement and appetite-related change scores were evaluated using Pearson correlation coefficients. Minimum detectable effects for the main SNAQ analyses were estimated at two-sided α = 0.05 and 80% power.

## 3 Results

A total of 26 patients were enrolled in the study, with a median age of 46.5 years, including 16 with MDD and 10 with BD (Table 1). Median disease duration was also comparable, at 8 years in the MDD group and 8.5 years in the BD group. Regarding medical comorbidities, hypertension was present in 15.4% of patients, diabetes mellitus in 7.7%, hyperlipidemia in 11.5%, and other medical conditions in 30.8%, while 57.7% of participants had no medical comorbidities. Oral ketamine was administered to 23 patients (88.46%). The mean dose was 2.13 mg/kg body weight (SD = 0.68). No significant differences in BMI were observed between the diagnostic groups. The median BMI was 26.59 kg/m^2^ in the overall sample, 25.85 kg/m^2^ in the MDD group, and 27.65 kg/m^2^ in the BD group (IQR: 22.76–29.54, 22.32–27.83, and 24.01–32.19, respectively). Notably, no participants in the BD group had a BMI below 18.5. Seven participants met the criteria for obesity, with comparable distribution across diagnostic categories: 42.9% in the MDD group and 57.14% in the BD group (Table 2). No clinically meaningful sex differences in the prevalence of obesity were identified, with a discrepancy of approximately 14%. A similar pattern was observed within the non-obese subgroup.

**TABLE 1:** Baseline demographic, clinical, and anthropometric characteristics of the study cohort.

Variable		MDD (*N* = 16)	BD (*N* = 10)	All (*N* = 26)
Age (years) [median (IQR)]		44.5 (29.5–58.25)	48.5 (35.5–56.25)	46.5 (30.5–57.75)
Disease duration (years) [median (IQR)]		8 (2.75–11.5)	8.5 (3.38–13.75)	8.5 (2.62–13.75)
Body mass (kg) [median (IQR)]		82 (62.75–93.75)	77 (69.88–87.5)	80 (65.12–91.47)
Height (m) [median (IQR)]		1.75 (1.68–1.81)	1.65 (1.6–1.73)	1.72 (1.61–1.81)
BMI (kg/m^2^) [median (IQR)]		25.85 (22.32–27.83)	27.65 (24.01–32.19)	26.59 (22.76–29.54)
Sex: male, *n* (%)		9 (56.2%)	2 (20%)	11 (42.3%)
Sex: female, *n* (%)		7 (43.8%)	8 (80%)	15 (57.7%)
Ketamine route	Oral	23 (88.46%)		
	Intravenous	3 (11.54%)		
Ketamine dose (mg/per kg)		2.13 (SD 0.68)		

**TABLE 2:** Demographic and clinical characteristics of the study group stratified by obesity status.

Variable	Obese (*N* = 7)	Non-obese (*N* = 19)
BMI (kg/m^2^) [median (IQR)]	31.64 (31.19–32.56)	24.76 (21.6–26.87)
Age (years) [median (IQR)]	51 (48.5–59)	43 (29–54)
Disease duration (years) [median (IQR)]	16 (7–22.5)	7 (2.25–10)
Sex: male/female, *n* (%)	3 (42.9%)/4 (57.1%)	8 (42.1%)/11 (57.9%)
Diagnosis: BD, *n* (%)	4 (57.1%)	6 (31.6%)
Diagnosis: MDD, *n* (%)	3 (42.9%)	13 (68.4%)

For the SNAQ assessment (Table 3), data from 23 patients were included: 16 with MDD and eight with BD. No statistically significant differences in appetite were identified before versus after ketamine treatment, either within diagnostic subgroups or between them. The proportion of participants at risk of losing at least 5% of body weight within 6 months was 33.33% in the MDD group and 37.5% in the BD group. The highest risk was observed among patients with obesity (50%); however, this result did not reach statistical significance.

**TABLE 3:** Comparison of Simplified Nutritional Appetite Questionnaire (SNAQ) scores and nutritional risk at baseline and after completion of ketamine treatment.

Group	*N*	SNAQ (before)	SNAQ (after)	*P*	Risk before *n* (%)*	Risk after *n* (%)*	*P*
MDD	15	15 (14–16.5)	15 (14–16)	0.323	5 (33.3%)	5 (33.3%)	1
BD	8	15 (13–16)	15.5 (11.5–16.25)	0.595	3 (37.5%)	3 (37.5%)	1
All	23	15 (14–16)	15 (14–16)	0.24	8 (34.8%)	8 (34.8%)	1
Obese	6	14.5 (11–15.75)	15 (12.5–16.75)	0.408	3 (50%)	3 (50%)	1
Non-obese	17	15 (14–16)	15 (14–16)	0.417	5 (29.4%)	5 (29.4%)	1

*Percentage of patients at risk of ≥5% body weight loss within 6 months. Median scores (IQRs) are presented.

When assessing appetite, hunger, and desire to eat in relation to ketamine treatment, the only statistically significant result was the value for “desire to eat after 24 h” in the BD group (*p* = 0.023); however, no other statistically significant differences were detected in the overall cohort or between diagnostic subgroups (Table 4). None of the measured variables showed meaningful change across time points.

**TABLE 4:** Comparison of Simplified Nutritional Appetite Questionnaire (VAS) ratings of appetite, hunger, and desire to eat at baseline and after ketamine administration in patients with MDD and BD.

	*N*	MDD			BD			All		
Measure and time point		Before	After	*P*	Before	After	*P*	Before	After	*P*
Appetite before administration	21	5 (4.72–5.7)	4.99 (4.3–5.75)	0.889	3.14 (1–4.68)	1.93 (0.52–4.6)	0.8	4.8 (3.7–5.6)	4.5 (1.56–5.5)	0.94
Appetite after 4 h	20	5.03 (4.3–8.1)	4.85 (3.53–8.53)	0.756	4.6 (3.07–5.54)	3.6 (2.66–7.02)	0.933	5 (3.25–7.38)	4.5 (2.95–7.88)	0.828
Appetite after 8 h	20	5 (4.68–5.49)	4.75 (3.9–5)	0.756	3.94 (1.14–5.57)	2.93 (2.01–5.41)	0.383	5 (2.66–5.49)	4.59 (2.69–5.04)	0.387
Appetite after 12 h	20	5.15 (4.7–5.3)	4.83 (3.9–5.1)	0.556	2.53 (0.76–4.5)	2.09 (0.78–5)	1	4.75 (2.22–5.28)	4.6 (2.04–5.18)	0.615
Appetite after 24 h	21	4.9 (4.7–5.3)	4.99 (4.72–6.2)	0.563	4.6 (2.99–6)	3.72 (2.43–6.68)	0.554	4.8 (4.35–5.9)	4.9 (3.65–6.3)	0.948
Appetite after 48 h	20	4.9 (4.7–5.2)	5.05 (4.89–5.5)	0.784	4.5 (1.3–6.8)	4.17 (2.15–5.81)	0.688	4.9 (4.5–5.3)	5.05 (4.1–5.72)	1
Appetite after 7 days	17	5.03 (4.65–6.06)	5 (4.34–5.71)	0.333	1.1 (0.9–1.87)	2.96 (0.2–4.6)	0.812	4.8 (1.87–5.95)	4.83 (2.96–5.21)	0.426
Hunger before administration	21	3.9 (2–5)	4.8 (3.6–5.2)	0.235	1.3 (0.77–3.78)	3.05 (1.38–6.72)	0.293	3.1 (1–5)	4.5 (1.77–6.3)	0.11
Hunger after 4 h	21	4.35 (2.05–7.4)	4.83 (3.2–8.35)	0.34	4.35 (3.22–5.12)	3.98 (2.39–4.6)	0.945	4.35 (2.09–6.1)	4.2 (2.53–5.9)	0.517
Hunger after 8 h	20	4.45 (1.24–4.92)	4.75 (3.9–5.09)	0.23	4.45 (2.36–6.72)	2.97 (1.85–6.03)	0.205	4.45 (1.48–5.56)	4.45 (2.58–5.28)	0.931
Hunger after 12 h	20	4.9 (2.2–5.4)	4.7 (4.2–5)	0.6	1.7 (1.25–2.51)	2.53 (0.75–4.35)	0.834	3.62 (1.32–5.39)	4.65 (2–5.03)	0.546
Hunger after 24 h	21	5.05 (4.45–6.6)	4.95 (4.6–6.2)	0.893	4.92 (0.7–6.38)	3.82 (2.11–5.38)	0.554	5.05 (2.1–6.6)	4.9 (3.65–6.2)	0.808
Hunger after 48 h	20	4.5 (2.2–5.1)	5.2 (4.9–5.9)	0.294	5.3 (1.1–5.95)	4.2 (2.28–5.71)	1	4.7 (1.9–5.38)	5.1 (4.19–5.95)	0.433
Hunger after 7 days	17	5 (4.79–5.52)	4.71 (3.8–5.45)	0.919	2.2 (1.43–5)	2.96 (0.2–4.4)	0.312	4.95 (2.2–5.3)	4.6 (2.96–5.11)	0.41
Desire before administration	20	5.03 (2.77–5.39)	4.97 (4.19–6.37)	0.791	3.15 (0.68–5.28)	3.18 (1.61–6.97)	0.383	4.95 (1.27–5.39)	4.87 (2.32–6.97)	0.388
Desire after 4 h	21	3.7 (2.6–8.13)	4.8 (3.5–8.5)	0.305	3.65 (0.89–6.08)	3.6 (2.63–4.65)	0.742	3.7 (1.1–6.6)	4.27 (2.81–5.8)	0.243
Desire after 8 h	20	4.45 (3–5.28)	4.87 (2.8–5.22)	0.85	4.26 (2.25–7.15)	3.07 (1.57–5.25)	0.313	4.45 (2.25–6.18)	4.47 (2.2–5.22)	0.43
Desire after 12 h	20	5 (3.6–5.3)	4.8 (3.3–5.3)	0.542	2.42 (1.48–5.05)	2.42 (0.88–5.1)	0.787	4.72 (1.49–5.38)	4.49 (2.31–5.57)	0.794
Desire after 24 h	21	5.2 (4.9–5.8)	5.2 (4.8–6.7)	0.497	6.05 (2.77–7.02)	3.09 (1.12–4.59)	0.023	5.3 (3.62–6.4)	4.99 (2.53–6.5)	0.393
Desire after 48 h	19	4.6 (3.9–5.4)	5.2 (4.5–6.3)	0.906	6.25 (3.67–8.15)	4.26 (1.88–5.54)	0.438	4.9 (3.5–6.5)	5.1 (3.53–6.18)	0.616
Desire after 7 days	17	4.92 (3.97–5.32)	5.1 (4.25–6.12)	0.241	2.2 (2.2–8.4)	2.85 (0.3–4.5)	0.125	4.9 (2.4–5.4)	4.94 (2.85–5.21)	0.733

Similarly, when comparing obese and non-obese patients, no statistically significant differences were found in any of the evaluated measures before or after ketamine administration (Table 5).

**TABLE 5:** Comparison of Visual Analog Scales (VAS) ratings of appetite, hunger, and desire to eat at baseline and after ketamine administration in obese and non-obese patients with treatment-resistant mood disorders.

	*N*	Obese			Non-obese		
Measure and time point		Before	After	*P*	Before	After	*P*
Appetite before administration	14	4.72 (0.81–5)	1.56 (0.7–4.83)	0.866	5 (4.08–5.68)	4.95 (2.95–7.44)	0.78
Appetite after 4 h	13	4.5 (2.74–4.93)	4.7 (2.96–5.93)	0.675	5.05 (4.7–7.9)	4.3 (3–8.4)	0.969
Appetite after 8 h	13	5 (0.98–6.24)	4.7 (1.79–5.24)	0.578	5 (4–5.1)	4.47 (3–5)	0.505
Appetite after 12 h	13	4.3 (0.76–4.99)	4.7 (1.01–5.67)	0.529	4.8 (4.25–5.3)	4.5 (2.8–5.1)	0.893
Appetite after 24 h	14	4.72 (2.57–5.4)	4.72 (2.15–5.65)	0.584	4.93 (4.41–6.16)	5 (4.02–6.27)	0.778
Appetite after 48 h	13	4.9 (1.58–5.17)	4.5 (2.36–5.34)	0.937	4.9 (4.7–5.27)	5.3 (4.89–6.2)	0.969
Appetite after 7 days	12	1.1 (0.9–4.8)	4.83 (0.44–5)	1	5.08 (4.2–6.51)	4.82 (3.29–5.71)	0.374
Hunger before administration	14	1 (0.05–4.05)	4.5 (1.22–5.62)	0.297	3.78 (1.7–5.07)	4.6 (2.55–7.3)	0.268
Hunger after 4 h	14	3.6 (1.04–4.4)	4.2 (2.92–4.84)	0.375	4.85 (2.96–7.09)	4.4 (2.69–8.24)	0.903
Hunger after 8 h	13	5.15 (0.22–6.95)	4.85 (1.69–5.48)	0.688	4.2 (2.6–4.85)	4.2 (3.3–5.2)	0.689
Hunger after 12 h	13	1.1 (0–2)	4.72 (0.96–5.05)	0.142	5.1 (2.42–5.5)	4.6 (2.53–5)	0.542
Hunger after 24 h	14	0.9 (0.33–5.45)	4.61 (2.04–4.95)	0.402	5.1 (4.46–6.6)	5.08 (4.15–6.27)	0.358
Hunger after 48 h	13	2.1 (0.66–5.2)	4.9 (2.36–5.29)	0.402	4.9 (4.2–6.3)	5.2 (4.2–6.4)	0.78
Hunger after 7 days	12	5 (1.43–5.05)	4.72 (0.66–4.9)	0.201	4.93 (4–6.38)	4.5 (3.37–5.45)	0.689
Desire before administration	13	4.9 (0.05–5.08)	4.8 (1.26–5.77)	0.375	5 (2.7–6.1)	5 (2.42–8.1)	0.787
Desire after 4 h	14	1 (0.27–3.65)	4.27 (3.01–4.95)	0.109	5.52 (3.14–7.87)	4.35 (2.6–8.32)	0.855
Desire after 8 h	13	5.25 (0–7.2)	4.75 (1.37–5.19)	0.578	4.3 (2.53–5.38)	4.2 (2.74–5.2)	0.635
Desire after 12 h	13	1.5 (0–4.25)	4.8 (0.95–5.83)	0.402	5.1 (3.6–5.6)	4.2 (2.7–5.3)	0.845
Desire after 24 h	14	5.2 (0.33–6.05)	3.65 (1.01–5.09)	0.375	5.38 (4.91–6.52)	5.15 (3.34–7)	0.715
Desire after 48 h	13	4.2 (1.33–5.62)	4.63 (1.88–5.25)	0.892	4.9 (4.45–6.7)	5.1 (4.45–6.3)	0.455
Desire after 7 days	12	4.94 (2.2–5.3)	4.7 (0.55–4.94)	0.201	4.8 (3.3–5.8)	5.1 (2.89–6.12)	0.625

**FIGURE 3:**
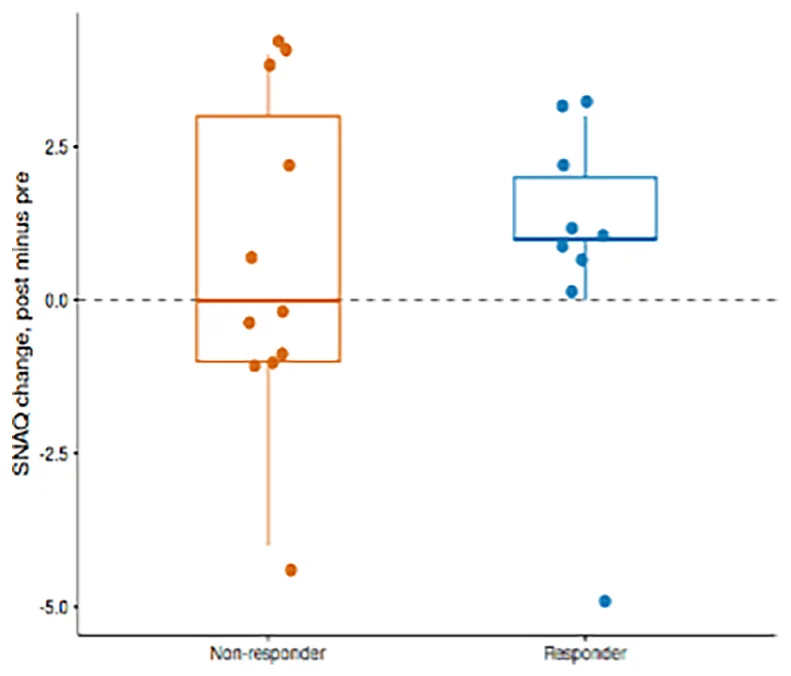
Simplified Nutritional Appetite Questionnaire (SNAQ) change scores in responders and non-responders. The boxplots display median values and interquartile ranges, with individual participant data overlaid. Positive values correspond to increased appetite ratings after treatment and negative values to decreased ratings.

**FIGURE 4:**
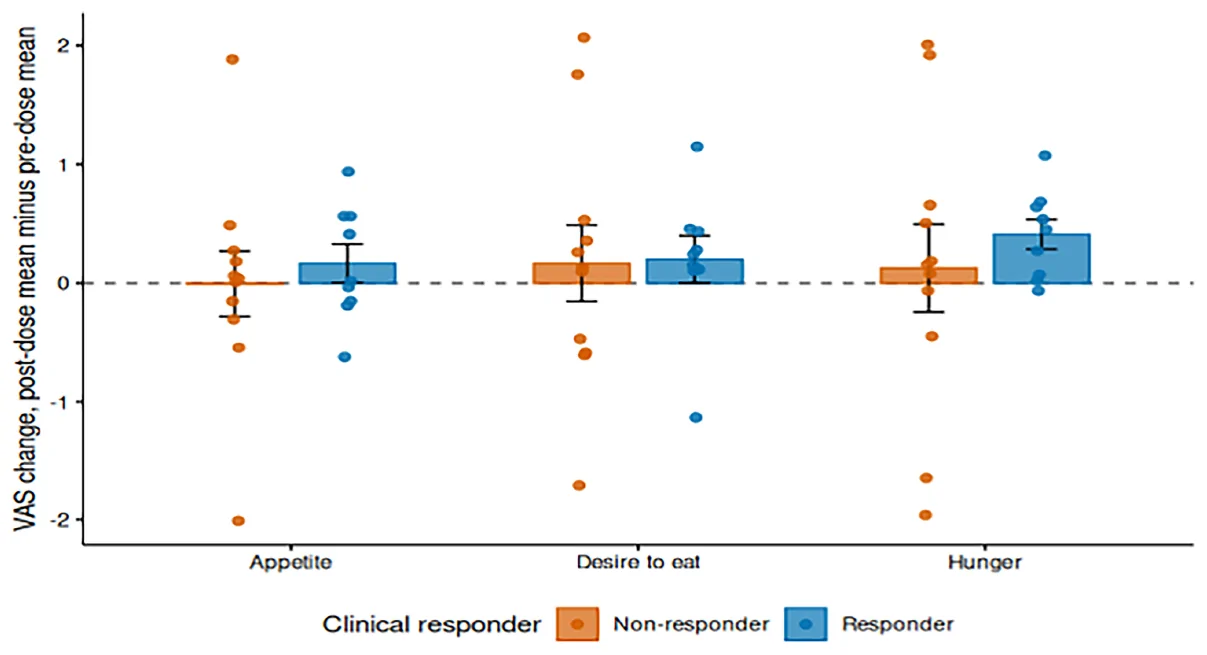
Changes in Visual Analog Scales (VAS)-rated appetite, desire to eat, and hunger according to clinical response. Individual observations are shown together with group means and confidence intervals. Positive values indicate increased ratings following treatment, whereas negative values indicate decreased ratings.

To examine whether appetite-related changes were associated with antidepressant response to ketamine, participants were classified as responders or non-responders based on a ≥ 50% reduction in MADRS scores from baseline to the last valid post-baseline assessment. Clinical response could be determined for 22 patients, of whom 10 were classified as responders and 12 as non-responders. Four patients could not be classified due to missing outcome data. Mean changes in SNAQ scores were similar in responders and non-responders, with no between-group difference detected (Cohen’s d = 0.02, *p* = 0.9643) (Figure 2 and Table 6). Mood improvement was not correlated with SNAQ change (*r* = 0.02, *p* = 0.9455). VAS ratings of appetite, hunger, and desire to eat were similar in responders and non-responders. No between-group differences were detected (Cohen’s d = 0.23, 0.30, and 0.04; *p* = 0.5948, 0.4814, and 0.9303, respectively). None of these measures was associated with improvement in depressive symptoms (appetite: *r* = −0.02, *p* = 0.9433; hunger: *r* = 0.05, *p* = 0.8330; desire to eat: *r* = −0.04, *p* = 0.8723) (Figures 3, 4 and Table 6).

**TABLE 6:** Changes in appetite-related measures by clinical response.

Measure	Responder	Non-responder	Complete cases	Mean difference	Cohen *d*	Welch *p*	Pearson *r*	*P*
SNAQ	*n* = 9; 0.78 (SD 2.39)	*n* = 11; 0.73 (SD 2.57)	9 vs. 11	0.05	0.02	0.9643	0.02	0.9455
Appetite (VAS)	*n* = 9; 0.17 (SD 0.49)	*n* = 11; −0.01 (SD 0.91)	9 vs. 11	0.17	0.23	0.5948	−0.02	0.9433
Hunger (VAS)	*n* = 9; 0.41 (SD 0.37)	*n* = 11; 0.13 (SD 1.23)	9 vs. 11	0.28	0.30	0.4814	0.05	0.8330
Desire to eat (VAS)	*n* = 9; 0.20 (SD 0.59)	*n* = 11; 0.17 (SD 1.07)	9 vs. 11	0.03	0.04	0.9303	−0.04	0.8723

Values are mean change (SD). Mean diff. is responder minus non-responder change.

A *post hoc* power analysis was conducted for the main SNAQ-related analyses (Table 7). With a complete-case sample of 23 participants, the paired SNAQ analysis had 80% power to detect an effect size of d = 0.61 (approximately 1.47 SNAQ points). For the responder versus non-responder comparison (*n* = 9 vs. 11), 80% power required a between-group effect size of d = 1.33 (approximately 3.32 SNAQ points). For the association between mood improvement and SNAQ scores, 80% power required a correlation coefficient of *r* = 0.57.

**TABLE 7:** Formal power analysis and type II error note.

Analysis	Complete-case sample	80% power threshold	Raw-scale note
Paired SNAQ	23	Cohen *d* = 0.61	About 1.47 SNAQ points
Responder SNAQ comparison	9 vs. 11	Cohen *d* = 1.33	About 3.32 SNAQ points
Mood/SNAQ correlation	20	Pearson *r* = 0.57	No raw-unit conversion

Power-analysis thresholds. Rows show complete-case samples and the effect required for 80% power at two-sided alpha = 0.05.

## 4 Discussion

In this naturalistic study, we examined the effects of ketamine treatment on appetite-related outcomes in patients with treatment-resistant mood disorders. Thus, the study found no increased risk of body weight loss or appetite-related undernutrition associated with ketamine therapy. Across repeated assessments, we observed no statistically significant changes in appetite, hunger, or desire to eat, either in the overall sample or after stratification by diagnosis (MDD vs. BD), mood improvement or body mass index (obese vs. non-obese). These parameters remained stable from treatment initiation through completion, indicating that ketamine did not meaningfully alter appetite-related outcomes at any stage of therapy. Importantly, no differential effects were detected between obese and non-obese participants, suggesting a consistent appetite-related response profile across BMI categories.

According to our previous systematic review (16), the available evidence regarding the effects of ketamine on appetite remains limited and heterogeneous. While some studies reported improvements in appetite, others found no significant changes, and one study observed a worsening of appetite despite a favorable antidepressant response. Taken together, these findings suggest that appetite-related symptoms may respond differently to treatment than other depressive symptoms.

**FIGURE 5:**
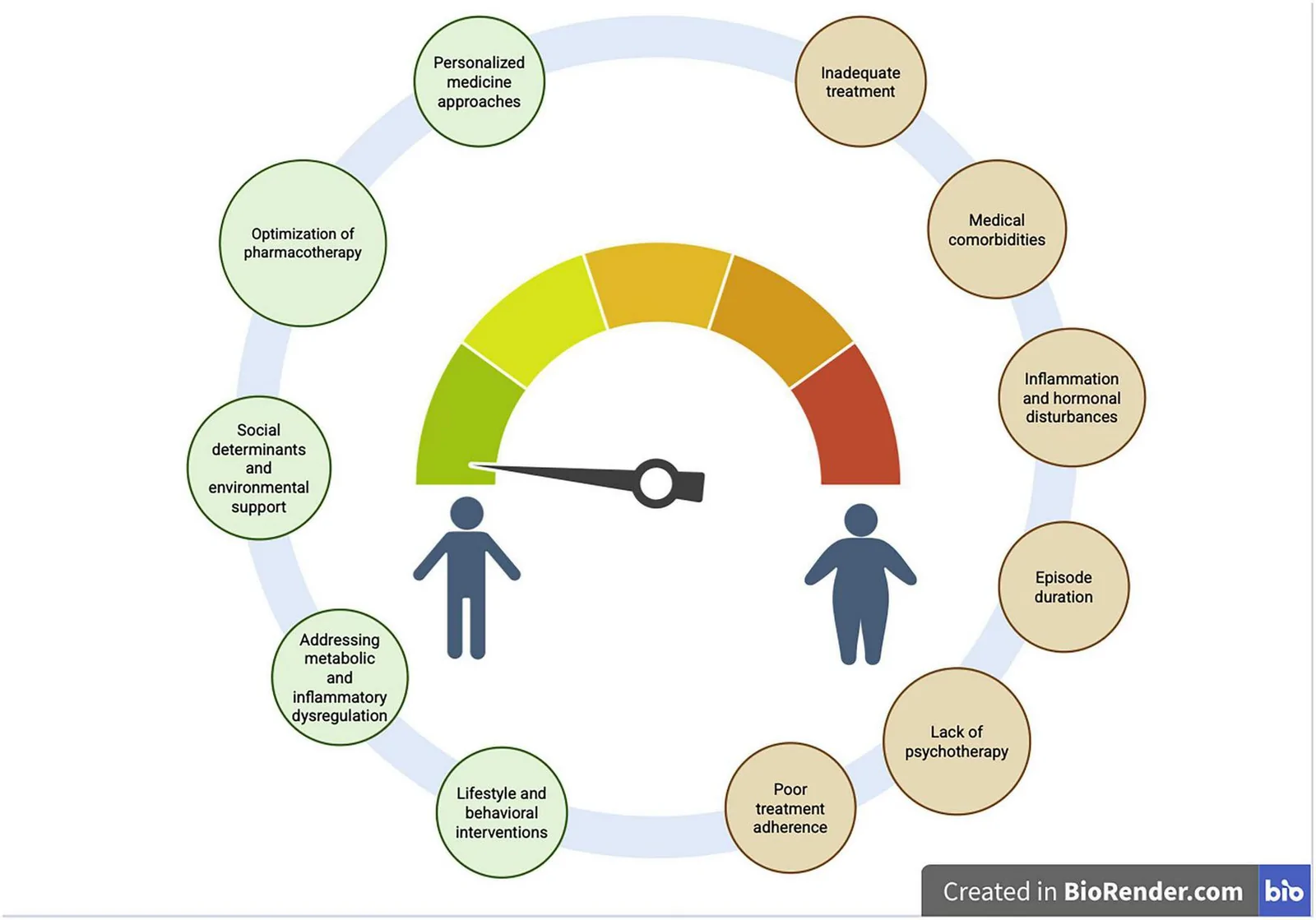
Conceptual scheme illustrating the relationship between treatment-resistant mood disorders and metabolic dysregulation. Individuals with affective disorders are at increased risk of obesity and metabolic disturbances, which may be further exacerbated by psychopharmacological treatment, particularly antipsychotics and mood stabilizers that influence appetite regulation, adipose tissue expansion, and glucose-insulin homeostasis (25). Weight gain and metabolic complications may, in turn, adversely affect treatment response and adherence, with metabolic side effects representing a major contributor to premature treatment discontinuation (27, 28). Optimized, stage-specific treatment strategies incorporating metabolically neutral pharmacotherapy, lifestyle modification, and targeted metabolic interventions may mitigate these risks and improve long-term outcomes in treatment-resistant mood disorders (29, 30). Created in https://BioRender.com.

Mood disorders are chronic conditions that extend beyond isolated central nervous system dysfunction and are closely intertwined with systemic metabolic and inflammatory processes (Figure 5). Excess body weight, metabolic disturbances, and the endocrine activity of adipose tissue interact bidirectionally with affective symptoms, amplifying overall disease burden (19). Prior research indicates that appetite changes in depression tend to follow relatively stable phenotypes, characterized by either hypo- or hyperphagia, underscoring the importance of depressive subtyping (20, 21).

In particular, atypical depression is more frequently associated with hyperphagia, weight gain, heightened inflammatory activity, and elevated leptin levels compared with melancholic presentations (20). Leptin was also investigated as a potential predictor of response to ketamine treatment. However, no significant association was found between leptin levels and treatment efficacy. A slightly better therapeutic response was observed in patients with higher BMI values, a characteristic that is generally associated with elevated circulating leptin concentrations (22). Against this background, the absence of measurable appetite changes in our cohort suggests that ketamine does not substantially influence these established appetite phenotypes. Although obese participants showed a non-significant tendency toward appetite reduction, this effect did not reach statistical significance. From a clinical perspective, the lack of appetite increase may be regarded as a favorable outcome in individuals with excess body weight, whereas patients with lower BMI appear unlikely to derive appetite-related benefit from ketamine, highlighting the importance of individualized nutritional monitoring. Our findings are broadly consistent with previous observations indicating that ketamine’s therapeutic effects are more pronounced in core depressive symptoms than in atypical features such as increased appetite or carbohydrate craving (23). While modest reductions in craving have been reported, ketamine’s overall impact on appetite appears limited, reinforcing the notion that mood improvement and appetite regulation may be governed by partially distinct neurobiological systems.

The prevalence of obesity is disproportionately high among individuals with psychiatric disorders, particularly those with bipolar disorder and schizophrenia, a pattern largely attributed to long-term exposure to psychotropic medications (24). Second-generation antipsychotics - especially olanzapine - are associated with profound metabolic effects due to their broad receptor-binding profiles, while mood stabilizers such as lithium and valproic acid may indirectly affect metabolic homeostasis via thyroid dysfunction or leptin signaling. Sex-related differences further modulate metabolic vulnerability, with women appearing particularly susceptible to treatment-induced metabolic syndrome (25). In this context, the absence of clinically meaningful changes in appetite-related measures in our cohort - regardless of diagnosis, BMI, or sex - suggests that ketamine is unlikely to promote clinically relevant disturbances in body weight and may exhibit a more favorable metabolic profile than many commonly used psychotropic agents. Although data on metabolic parameters during ketamine treatment remain limited, available evidence indicates no disruption of glucose–insulin homeostasis in adults with MDD. Ketamine may nonetheless indirectly influence metabolic pathways, for example through modulation of adipokines such as adiponectin, which has been proposed as a marker of bipolar disorder progression (19). No significant differences were observed between MDD and BD groups across any appetite-related parameters. This finding aligns with evidence that metabolic abnormalities are prevalent across mood disorders and may contribute to a more severe clinical course when coexisting with depression (5). Obesity has been linked to bipolar spectrum features and mixed symptoms, suggesting that excess body weight may function as a clinical marker warranting increased diagnostic vigilance (26). Moreover, BMI correlates positively with age and adverse metabolic profiles, including elevated triglycerides, LDL cholesterol, HbA1c, and hsCRP, emphasizing the need for systematic metabolic screening and proactive weight-management strategies (24). Taken together, the apparent lack of ketamine-related metabolic disruption—particularly weight gain-related effects - even among obese patients supports its metabolic safety and favors its use in treatment-resistant mood disorders.

This study has several limitations. The most important include the relatively small sample size, the short observation period, and the inpatient setting. The limited sample size also reduced the ability to identify subtle appetite-related effects. Power calculations indicated that the available sample was sufficient to detect moderate-to-large effects in the paired SNAQ analysis and large effects in responder versus non-responder comparisons. Therefore, smaller changes in appetite may have remained undetected, and the absence of significant associations between appetite measures and antidepressant response should be interpreted with caution. It should be noted that ketamine administration required hospital-based procedures, and the study followed a naturalistic observational design. Moreover, recruitment could only occur when patients were already hospitalized for clinical reasons, precluding the possibility of inviting independent outpatients and constraining the final sample composition in terms of gender distribution and diagnostic subgroups (MDD vs. BD). Although the closed-ward environment partially standardized access to meals, patients still had the possibility of consuming various hospital diets as well as personal snacks, which may have influenced appetite regulation. Importantly, to the best of our knowledge, this is the first original study to evaluate the impact of ketamine treatment on appetite, its fluctuations, and the potential metabolic risks related to body weight in patients with treatment-resistant mood disorders. Moreover, the study employed validated assessment tools - VAS and SNAQ - both of which demonstrate strong reliability. These findings have implications for both research and clinical practice. Ketamine treatment appears metabolically neutral with respect to appetite and body weight in the short term, supporting its safety profile in patients with treatment-resistant mood disorders. Further research in larger samples is needed to clarify the long-term metabolic effects of ketamine and its impact on nutritional status.

## 5 Conclusion

In this naturalistic study, ketamine treatment was not associated with measurable changes in appetite, hunger, or desire to eat among patients with treatment-resistant mood disorders, regardless of diagnosis, mood improvement or BMI category. Appetite-related measures remained stable throughout the entire treatment period. Taken together, these findings do not suggest a substantial short-term impact of ketamine on appetite regulation or body weight and are consistent with a favorable metabolic safety profile, supporting its clinical use in patients with TRD and TRBD. Nevertheless, ongoing metabolic and nutritional monitoring remains essential - particularly in metabolically vulnerable subgroups - as part of a comprehensive and individualized treatment strategy.

## Data Availability

The raw data supporting the conclusions of this article will be made available by the authors upon reasonable request.
